# Transcriptomic Analysis Suggests Shoots and Roots-Specific Antioxidant Responses to Early-/Long-Term Salt Stress in *Brassica napus*

**DOI:** 10.3390/antiox15060708

**Published:** 2026-06-03

**Authors:** Xianmin Meng, Lei Lei, Weirong Wang, Hongwei Li, Jifeng Zhu

**Affiliations:** Key Laboratory of Germplasm Innovation and Genetic Improvement of Grain and Oil Crops (Co-Construction by Ministry and Province), Ministry of Agriculture and Rural Affairs, Key Laboratory of Agricultural Genetics and Breeding of Shanghai, Crop Breeding and Cultivation Research Institute, Shanghai Academy of Agricultural Sciences, Shanghai 201403, China; mengxianmin@saas.sh.cn (X.M.); leilei@saas.sh.cn (L.L.); 20150230@saas.sh.cn (W.W.); lihongwei@saas.sh.cn (H.L.)

**Keywords:** *Brassica napus*, salt stress, Na^+^/K^+^, antioxidant enzymes, transcriptomics

## Abstract

Salinity stress progressively restricts rapeseed (*Brassica napus* L.) growth and productivity. However, the molecular mechanism underlying its tolerance remains poorly understood. This study aims to shed light on differential responses between shoots and roots, and further clarify the regulatory mechanisms of ion homeostasis and oxidative defense under early-and long-term salt stress. Under salt stress, the Na^+^/K^+^ ratio increased by 46.26% and 26.33% in shoots and roots, respectively. Activities of SOD and POD increased in both tissues, while CAT activity declined in shoots. MDA content was significantly higher in roots. Transcriptome PCA clearly separated samples of early-term (3–48 h for shoots, 3–24 h for roots) from long-term (72 h 25 d for shoots, 48 h 25 d for roots) salt stress. *SOD2* and *UGT72E1* were significantly up-regulated in shoots but down-regulated in roots. *CAT2* exhibited strongly up-regulation in roots than shoots, whereas *RBOHC* was markedly down-regulated in roots relative to shoots. Additionally, *CAT1* was mainly up-regulated at the early-term salt stress. Most DEGs involved in phenylpropanoid biosynthesis (*CYP73A5*, *PAL2*, *CCR1*/*2*, *CAD1*/*5*, *COMT1* and *PER66*) were up-regulated in both tissues. Notably, *HCT* and *CSE* exhibited a striking tissue-specific antioxidant pattern, down-regulated in shoots but up-regulated in roots. *PER34* was specifically induced at early-term, and *PER31*/*63*/*169* were exclusively activated under long-term salt stress in roots. Moreover, we performed weighted gene co-expression network analysis (WGCNA) to describe tissue- and time-specific transcriptional dynamics that occur in rapeseed under salt stress. Several hub genes, including *ABI5*, *MPK6*, *CAD5*, *NADK1* and *LFG2*, exhibited high correlations with early-term salt stress responses in roots. These genes are mainly enriched in transcription factors and hormone signaling pathways, and function in antioxidant defense and redox homeostasis. This study suggests distinct spatiotemporal salt stress response patterns in rapeseed and identifies key genes for salt-tolerance breeding.

## 1. Introduction

Soil salinity is a key abiotic constraint limiting the growth performance and yield sustainability of oilseed crops across many regions of the world. It has been estimated that salinity affects approximately 7% of the land mass worldwide, or 950 million hectares [1]. Annually, approximately 1.5 million hectares cannot be farmed owing to excessive salt content in soils [2].

However, China’s large population base means that non-saline land resources are already under intense pressure, making it challenging to expand crop cultivation in these areas. Alleviating salt damage to crops requires in-depth research on salt adaptation mechanisms, which helps promote the development of salt-tolerant crop germplasm [3]. Salt stress-resistant crop breeding contributes to global food supply growth via improved land utilization and yield performance on saline soils.

Complex adaptive systems develop in plants to resist salt damage, including ion homeostasis maintenance, osmotic adjustment, antioxidant activation, hormone and gene regulation, and coordinated growth and stress tolerance [4]. Also, plants relieve sodium ion toxicity in the cytoplasm by expelling Na^+^ through high-affinity K^+^ transporter (*HKT1*) and salt overly sensitive 1 (*SOS1*), or compartmentalizing Na^+^ into vacuoles via Na^+^/H^+^ exchanger (*NHX1*) [5]. Compared with single Na^+^ content, the Na^+^/K^+^ ratio better reflects salt adaptability, which stays low in salt-tolerant plants to avoid salt toxicity as a defense and antioxidant system [6,7].

When plants perceive salt stress signals, elevated calcium ion (Ca^2+^) concentration acts as the first messenger to activate Ca^2+^ sensors and downstream pathways, such as SOS and mitogen-activated protein kinase (MAPK). Abundant active oxygen species (ROS) generated under saline conditions lead to oxidative injury, accompanied by ionic disorder, genetic lesion, biomolecule oxidation, protein deterioration, pigment degradation and abnormal enzyme activity [8,9].

In addition, antioxidant protection involves enzyme molecules, like superoxide dismutase (SOD), peroxidase (POD), and catalase (CAT), alongside nonenzymatic antioxidants including glutathione, ascorbic acid, flavonoids, carotenoids, phenolic compounds, and tocopherol [6,10]. This system is activated to scavenge excessive ROS and prevent ROS-mediated damage.

Phytohormone-mediated signaling pathways also enhance plant adaptive growth and salt stress resistance. Additionally, as transcription factors, the overexpression of *BnBBX22.A07* increased salt stress tolerance by activating the expression of ROS-scavenging-related genes, but also by indirectly activating *BnWRKY33.C03* in response to salt stress [11]. Loss of all six *BnaWIP2* homologous genes in *B. napus* markedly suppressed seed germination, and overexpression of *BnaC06.WIP2* boosted this process, which was induced by abscisic acid (ABA) and in turn restrained ABA production under salinity stress [12].

Rapeseed, *Brassica napus* L. (Brassicaceae, 2*n* = 38) is a major oil crop and shows promising value in saline–alkali improvement. It has wide applications, covering food, feeding, fertilization, honey production, biofuel and landscape planting [13,14]. Cultivating salt-resistant rapeseed on coastal saline lands, particularly in Shanghai’s Fengxian and Chongming districts, can expand local rapeseed production, relieve land resource constraints, and lower reliance on imported oil. Hence, exploring salt-tolerance mechanisms is vital for saline land remediation and sustainable agriculture.

Few genes and QTLs associated with salinity adaptation have been reported in rapeseed so far [15]. Experimental evidence confirms *BnCKX5* and *BnERF3* modulate salt tolerance in *B. napus* [16]. Salt stress triggers *BnaGRP3* expression, which further enhances salt tolerance by regulating the expression of ion transporters [17]. *ORF188* overexpression improved salt tolerance in rapeseed by enhancing growth, mitigating oxidative damage, maintaining a favorable Na^+^/K^+^ ratio and orchestrating a transcriptome tailored for stress homeostasis [2].

Furthermore, previous studies were conducted to uncover key metabolic variations in rapeseed cultivars during seed germination [18]. RNA-seq enables comprehensive detection of stress-induced gene expression changes and regulatory mechanisms [19], including key modules involved in hormone signaling, antioxidant defense, intracellular salt transport, and cell wall reinforcement [20]. Mining salt-tolerant genes and understanding their underlying molecular mechanisms under salinity is particularly important for the cultivation of salt-tolerant rapeseed.

However, the molecular mechanism underlying tissue- and stage- specific salt stress response in rapeseed remains unclear. Here, we performed a comprehensive physiological and transcriptomic analysis in shoots and roots across early- and long-term salt stress to elucidate the associated pathways, hub genes, and regulatory mechanisms. These findings enrich relevant theoretical knowledge and bring new perspectives on rapeseed salt-tolerance regulatory networks.

## 2. Materials and Methods

### 2.1. Plant Material, Cultivation, and Stress Treatment

*B. napus* accession 3506 was cultivated and harvested at the research base of the Shanghai Academy of Agricultural Sciences (30.88° N, 121.38° E, Shanghai, China). This germplasm is preserved in the germplasm bank of the Shanghai Academy of Agricultural Sciences. After three weeks of room temperature drying, the seeds were applied to salt tolerance tests. The salt-tolerant accession 3506 is characterized as a double-low rapeseed line with black seeds and belongs to the semi-winter ecotype.

For seedling experiments, sterilization was conducted using 75% alcohol (*v*/*v*) for 2 min and 1% NaClO (*w*/*v*) for 10 min, washed with ultrapure water for 5 min, and then sown on 1/2 MS medium. Three-day-old seedlings were fixed with cotton plugs and transferred to 10 L of half-strength Hoagland nutrient solution (NS10205, Coolaber, Beijing, China) for hydroponic culture, leaving approximately 1 cm of roots exposed for aeration. After two days, seedlings were transferred to full-strength Hoagland nutrient solution.

For salt stress treatment, three-leaf-stage (approximately three weeks post-germination) rapeseed plants were transferred to fresh Hoagland nutrient solution containing 275 mM NaCl or maintained in full-strength Hoagland nutrient solution as mock conditions.

The nutrient solution was replaced every three days. After 25 days of salt treatment, the whole aboveground shoots and underground roots were harvested for the determination of biomass and ion contents. An amount of 3 biological replicates were performed with 5 plants per replicate, and at least 30 plants were used for biomass and ion concentration determination. All plants were grown in a constant temperature cultivation room at 22 °C under a 16 h light/8 h dark photoperiod with a light density of 300–320 μmol m^−2^ s^−1^.

### 2.2. Measurement of Antioxidant Enzyme Activities in Rapeseed Shoots and Roots

Whole shoot and root samples at 3 h, 6 h, 12 h, 24 h, 48 h, 72 h and 25 days under control and NaCl treatment were collected, weighed (0.1 g) and extracted with 1 mL of extraction buffer containing 50 mmol L^−1^ phosphate buffer (pH 7.8) and 1% polyvinylpyrolidone. Enzyme activities were determined using SOD (AKAO001), POD (AKAO005), and CAT (AKAO003) detection kits (BOXBIO, Beijing, China) [21].

### 2.3. Measurement of MDA Content

For measuring malondialdehyde (MDA), 0.1 g of the whole shoots and roots samples collected at 3 h, 6 h, 12 h, 24 h, 48 h, 72 h and 25 days under control and NaCl treatment were ground in liquid nitrogen, adding 1 mL of 10% trichloroacetic acid (TCA) for extraction, and the concentration was determined according to the manufacturer’s instructions with the test kit (AKFA013, BOXBIO, Beijing, China).

### 2.4. RNA-Sequencing (RNA-Seq), Data Analysis and RT-qPCR

Whole shoot and root samples were sampled at designated time points (0 h, 3 h, 6 h, 12 h, 24 h, 48 h, 72 h and 25 days) for total RNA extraction, respectively. Three independent biological replicates were included per time point, with each replicate consisting of pooled tissues from at least five individual plants.

RNA extraction was performed using TRIzol (15596-026, Invitrogen, Carlsbad, CA, USA) following the manufacturer’s instructions, with residual DNA removed using RNase-free DNase I. The purity, concentration, and integrity of the RNA were assessed to ensure they met the prerequisites for library construction. Then, the libraries underwent sequencing on an Illumina HiSeq 4000 platform (Illumina, San Diego, CA, USA).

The Westar genome served as a reference genome. A local BLASTP, version 2.13.0, program was utilized to identify *Arabidopsis thaliana* (L.) (Brassicaceae) orthologs of each *B. napus* gene. Gene ontology (GO) analysis was conducted in DAVID Bioinformatics Resources (https://davidbioinformatics.nih.gov/ (accessed on 7 December 2025)). Differentially expressed genes (DEGs) defined as genes that met both the |log_2_ Fold Change| ≥ 2 and false discovery rate (FDR) ≤ 0.01 criteria in at least 1–2 of seven consecutive time points (both early-and long-term stress) or in either shoots or roots are listed in Tables S1 and S2. Clean RNA-Seq reads were deposited in the NCBI database (PRJNA1449748).

To verify the accuracy of RNA-seq data, nine genes were selected for RT-qPCR assay. cDNA libraries were constructed using a TranScript^®^ All-in-One First-Strand cDNA Synthesis Kit (AT341, Transgen, Beijing, China). qPCR was performed using a TransStart^®^ Top Green qPCR SuperMix Kit (AQ131, Transgen, Beijing, China) on a QuantStudio™ 6 Flex (Thermo Fisher Scientific, Basel, Switzerland).

The relative transcript abundance was calculated using the comparative *C*t method by 2^−ΔΔ*C*t^ [22]. The *BnaACTIN7* (*BnaC02G0037200ZS*) was used as an internal control. The primer information is listed in Table S3. Three biological replicates were performed per gene, and three technical replicates were performed within an experiment.

### 2.5. Gene Co-Expression Network Analysis (WGCNA)

In order to reduce noise, genes with low abundance and low variability were filtered out. A set of 25,536 variable genes was obtained by setting the threshold of the mean TPM value > 1 and the coefficient of variation < 0.1. First, the Soft-Threshold function was used to choose a soft power value by applying the approximate Scale-free Topology Criterion. We produced the scale-free topology fitting indices for different powers.

A suitable power was chosen when the scale-free topology fit did not improve after increasing the power, and with a signed R^2^ threshold > 0.9. Here, the value was 5. We then used the automatic network construction function block-wise modules to obtain weighted co-expression clusters, called modules, with the following settings for the calculation processes: power = 5, networkType = signed, corType = Pearson, minkMEtoStay = 0.3, minModuleSize = 30, and mergeCutHeight = 0.25.

We used tissue type and stress stage as the core trait variables for module-trait correlation analysis. Hub genes are those that show the most connections in the network as indicated by the high degree. The network of each module was exported to Cytoscape v3.10.3. The top 20 nodes ranked by ‘degree’, which was calculated by CytoHubba, were selected as hub nodes of the module [23].

### 2.6. Biological Function and Gene Set Enrichment Analyses

To investigate the biological functions of the differentiation-related modules, we performed enrichment analysis, including gene ontology (GO) term and Kyoto Encyclopedia of Genes and Genomes (KEGG) pathway enrichment analyses. Functional GO enrichment analysis was conducted using Goatools (https://github.com/tanghaibao/goatools (accessed on 7 December 2025)), whereas KEGG pathway enrichment analysis was carried out using KOBAS-i.

### 2.7. Determination of Na^+^ and K^+^ Concentrations

The whole shoots and roots of rapeseed seedlings were harvested separately, and Na^+^ and K^+^ concentrations were determined according to the methods described by Wang et al. [24]. Briefly, after drying at 80 °C for 2 days until constant weight was achieved, a 100 mg sample was digested with 5 mL of nitric acid at 90 °C for 8 h for digested completely. The mixture was diluted to 25 mL with distilled water after cooling it down. Na^+^ and K^+^ content analysis was carried out with an inductively coupled plasma-optical emission spectrometry instrument (ICP-OES, PerkinElmer, Waltham, MA, USA).

### 2.8. Statistical Analysis

All experiments were performed with a minimum of three biological repetitions (n = 5). The significance of differences was examined using one-way analysis of variance (ANOVA) with Tukey’s HSD test or two-tailed two-sample Student’s *t*-test by SPSS 20.0 (IBM Inc., Armonk, NY, USA), and *p* < 0.05 was considered significant. TBtools, version 2.476 [25] was applied to draw heat maps of pathway-related DEGs, and physiological data were visualized via Microsoft Excel. All data were expressed as the mean ± standard error (SEM) of replicates, was assessed using GraphPad Prism 8.0.

## 3. Results

### 3.1. Effect of Salt Stress on the Physiological Parameters of Rapeseed Shoots and Roots

To investigate the response of rapeseed shoots and roots to salt stress, 15-day-old seedlings were hydroponically grown in the presence of 275 mM NaCl for 3 h, 6 h, 12 h, 24 h, 48 h, 72 h and 25 days. Our previous work identified 136 *B. napus* germplasms at the germination stage and proved that 275 mM NaCl was the limiting concentration for germination of salt-tolerant rapeseed [26]. Seed germination is the most salt-sensitive stage, while rapeseed seedlings have stronger stress adaptability. Therefore, we selected 275 mM NaCl rather than setting multiple salt gradients, which is sufficient to efficiently distinguish salt tolerance variations in rapeseed.

**Figure 1:**
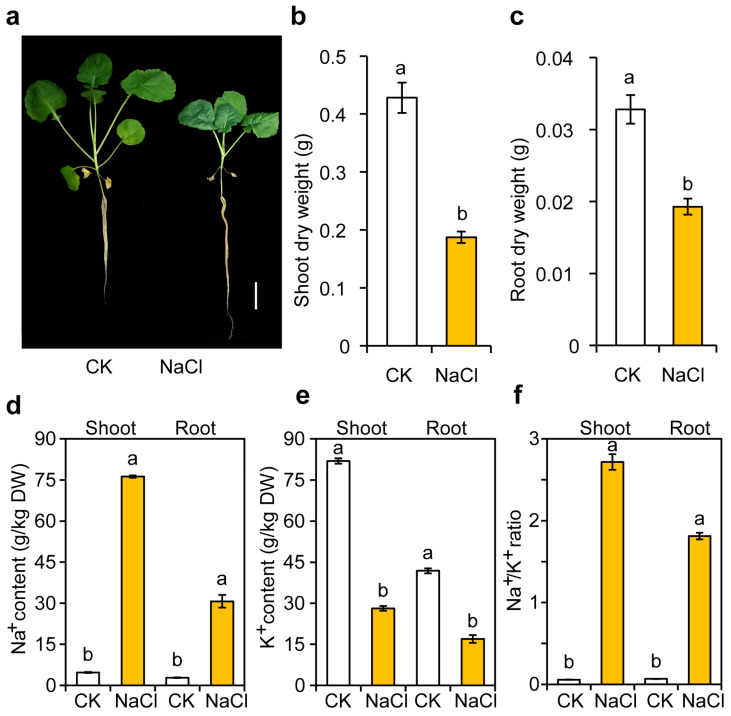
Phenotypes of salt stress response in rapeseed seedlings. (**a**) 15-day-old hydroponically grown seedlings were treated with 275 mM NaCl for 7 days. (**b**,**c**) Dry weight of shoots (**b**) and roots (**c**) in rapeseed seedlings under control and salt stress treatment, respectively. (**d**–**f**) Na^+^ content (**d**), K^+^ content (**e**) and Na^+^/K^+^ ratio (**f**) of shoots and roots in rapeseed seedlings under control and salt stress treatment. Bar: (**a**) 5 cm. The results in (**b**–**f**) are means ± standard error of three independent experiments. Statistical differences were denoted by different letters, as determined by a two-sided *t*-test (*p* < 0.05).

The weight and Na^+^, K^+^ content in both shoots and roots, as well as the antioxidant enzyme activities, were measured. Exposure to salt caused substantial declines in fresh biomass of plant tissues (Figure 1a). Shoots and roots exhibited 56.26% and 41.19% dry weight reduction (Figure 1b,c). Plants rely on steady Na^+^/K^+^ balance to tolerate saline habitats. More sodium was accumulated in shoots after salt stress, accompanied by reduced root potassium, driving the Na^+^/K^+^ ratio up by 46.26% and 26.33% in two tissues separately (Figure 1d–f).

**Figure 2:**
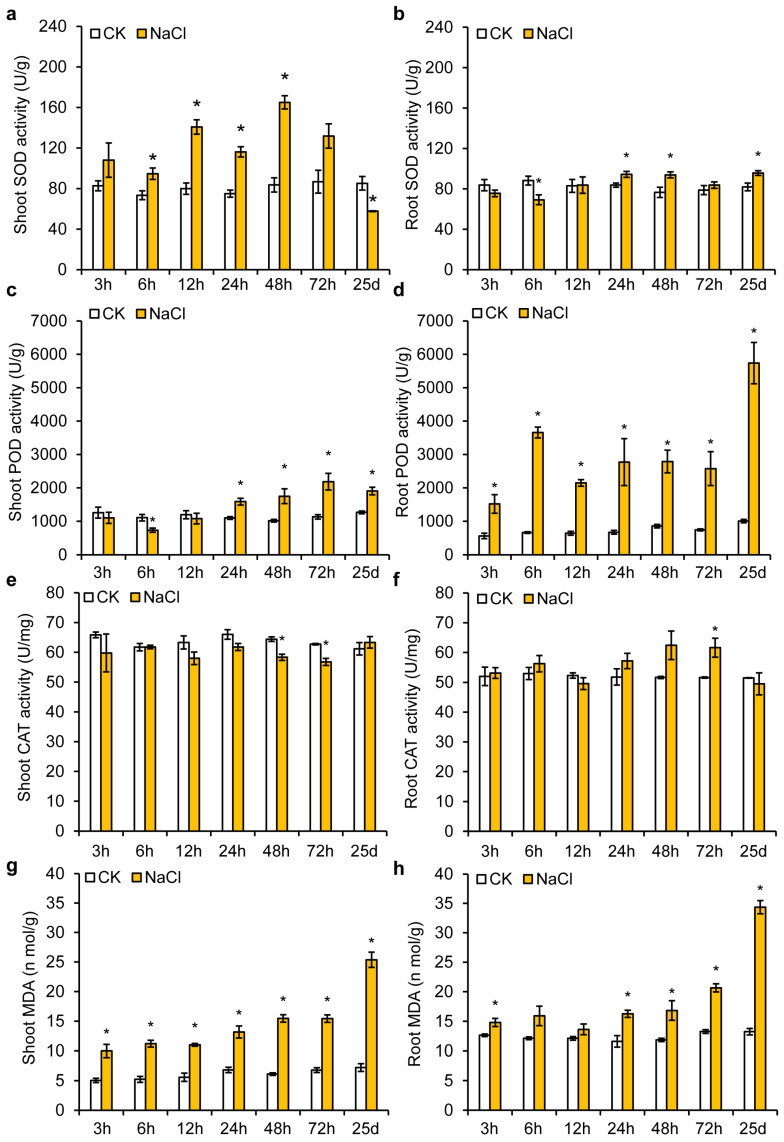
Physiological parameters of salt stress response in rapeseed at different time points. (**a**–**h**) Activities of superoxide dismutase (SOD) (**a**,**b**), peroxidase (POD) (**c**,**d**), catalase (CAT) (**e**,**f**) and content of malondialdehyde (MDA) (**g**,**h**) of shoots and roots in rapeseed seedlings under control and salt stress treatment at 3 h, 6 h, 12 h, 24 h, 48 h, 72 h and 25 days, respectively. The results in (**a**–**h**) are means ± standard error of three independent experiments. Statistical differences were denoted by asterisk, as determined by a two-sided *t*-test (*p* < 0.05).

In addition, SOD activity in shoots and roots showed an increasing trend, with significant increases observed at 24 h and 48 h compared to the respective controls under salt treatment (Figure 2a,b). POD activity was also significantly elevated in the shoots and roots of rapeseed s subjected to salt stress compared to controls (Figure 2c,d). Salinity suppressed shoot CAT activity, and the reduction intensified as stress duration increased (Figure 2e). In contrast, roots maintained a relatively higher activity level compared to the control (Figure 2f). The roots of rapeseed exhibited higher MDA content compared to shoots under salt treatment (Figure 2g,h).

Direct comparison of shoot and root tissues revealed significant tissue-specific differences in SOD, POD, CAT, and MDA levels at both 12 h and 25 d under salt stress (Figure 2). Ion accumulation, enzyme activity and gene expression all revealed distinct tissue responses to salt stress compared with control conditions.

### 3.2. Transcriptional Variations and Gene Expression Patterns in Rapeseed Shoots and Roots

RNA-seq on the Illumina platform was conducted for transcriptome analysis. A total of forty-eight cDNA libraries were constructed and full-length transcriptome sequencing was performed using the shoots and roots at eight time points (0 h, 3 h, 6 h, 12 h, 24 h, 48 h, 72 h and 25 days) after 275 mM NaCl treatment. Filtering out adapter sequences and low-quality reads yielded 320.16 Gb of valid data (Table S1). After quality trimming, the reads were aligned to the *B. napus* Darmor-*bzh* reference genome. The results showed that mapped reads accounted for 81.07–86.69% (Table S2).

**Figure 3:**
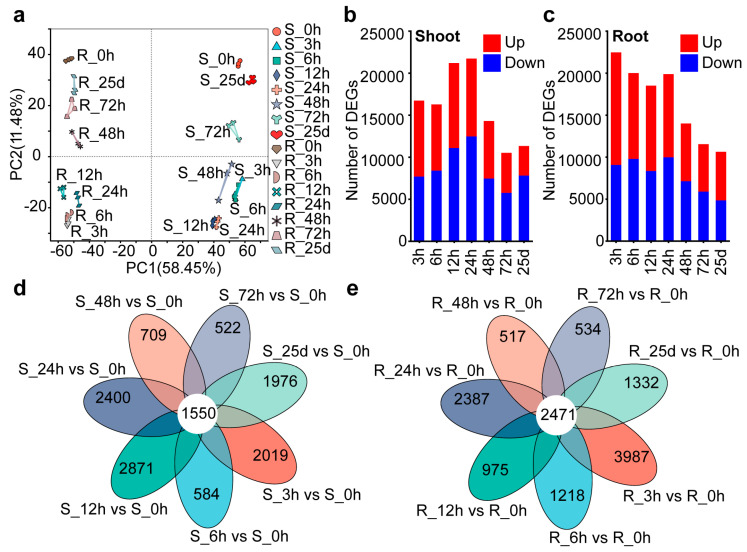
Multivariate statistical analysis of transcriptome data of each sample under salt stress in rapeseed shoots and roots. (**a**) PCA. Sample similarity is represented by point distance; closer points indicate higher similarity (dashed line at position 0). (**b**,**c**) Numbers of DEGs in different times of shoots (**b**) and roots (**c**) compared to their control (0 h). Red and blue columns represent the number of up-regulated and down-regulated genes, respectively. (**d**,**e**) Venn diagram showing the overlap of up-regulated genes (**d**) and down-regulated genes (**e**) at various time points. Colored circles indicate different gene sets.

Pearson correlation analysis showed intra-group R over 0.9, indicating high reproducibility of the biological replicates (Figure S1). Principal component analysis (PCA) indicated high repeatability of samples within groups and significant differences among early-term salt treatment (3, 6, 12, 24 and 48 h in shoots; 3, 6, 12 and 24 h in roots), long-term salt treatment (72 h and 25 days in shoots; 48 h, 72 h and 25 days in roots), and control (Figure 3a). Principal component 1 (PC1), explaining 58.45% of the total standing variation, distinguished the shoots and roots, while PC2, explaining 11.48% of the total variation, separated the early-and long-term salt treatments.

Differentially expressed genes (DEGs) (|log_2_ FoldChange| ≥ 2 and a *p*-adjust < 0.05) analysis revealed that, at 3, 6, 12, 24, 48, 72 h and 25 d after salt treatment, compared with the 0 h, 9028, 7870, 10,099, 9259, 6842, 4754 and 3507 up-regulated genes, 7694, 8402, 11,089, 12,466, 7455, 5758 and 7813 down-regulated genes were identified in shoots (Figure 3b), 13,382,10,214, 10,149, 9902, 6871, 5641 and 5773 up-regulated genes, 9067, 9772, 8340, 9966, 7117, 5883 and 4846 down-regulated genes were identified in roots (Figure 3c).

A total of 203 and 101 genes showed significantly up-regulated and down-regulated expression at all time points in shoots and roots after salt stress treatment, respectively (Figure 3d,e). KEGG enrichment analysis was performed on both common and specific DEGs to clarify functional differences. The DEGs between shoots and roots were also enriched in starch and sucrose metabolism, plant hormone signal transduction and phenylpropanoid biosynthesis.

**Figure 4:**
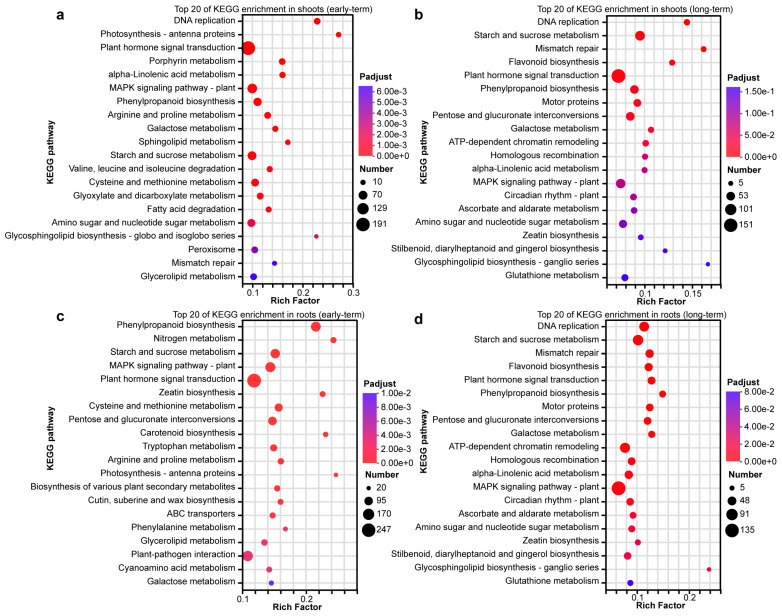
KEGG enrichment analysis of DEGs of each sample under salt stress in rapeseed shoots and roots. (**a**,**b**) KEGG enrichment analyses of early- (3, 6, 12, 24 and 48 h) (**a**) and long-term (72 h and 25 d) (**b**) salt treatment in shoots compared to 0 h, respectively. (**c**,**d**) KEGG enrichment analyses of early- (3, 6, 12, 24 h) (**c**) and long-term (48, 72 h and 25 d) (**d**) salt treatment in roots compared to 0 h, respectively. The vertical axis corresponds to functional pathways, while the horizontal axis indicates the enrichment factor. The dot size is proportional to the gene number enriched in each pathway, and the dot color denotes the adjusted *p* value.

In contrast, the DEGs between early-and long-term salt treatment were separately enriched in the MAPK signaling pathway and flavonoid biosynthesis, indicating that salt stress-related genes may regulate the tolerance of rapeseed to salt through these pathways (Figure 4a–d). Notably, the enrichment of the peroxisome pathway was observed specifically in shoots under early-term salt stress (Figure 4a), while tryptophan metabolism was uniquely enriched in roots at the same stage (Figure 4c), indicating tissue-specific early adaptive strategies in response to salt stress in rapeseed.

Gene ontology (GO) enrichment analysis illustrated that DEGs induced by early- and long-term salt stress in both shoots and roots were predominantly enriched in biological processes, with far fewer genes annotated in molecular function and cellular component terms (Figure S2a–d). For the DEGs that continuously increased and decreased in early-term salt stress in shoots, the most enriched terms from the ‘biological processes’ category were the response to water deprivation and DNA replication initiation, while the most enriched terms were the response to temperature stimulus and cell cycle process of long-term salt stress (Figure S3a–d).

For the DEGs that continuously increased and decreased during early-term salt stress in roots, the most enriched terms from the ‘biological processes’ category were the response to oxygen-containing compound and cell wall organization biogenesis, while the most enriched were the response to oxygen-containing compound and carbohydrate metabolic process of long-term salt stress (Figure S3e–h). These results suggested that the expression pattern of oxygen-containing compound, plant hormone signal transduction and phenylalanine metabolism genes is involved in coping with salt stress.

### 3.3. Categories of Genes Responsive to Salt Stress at Different Time Points

**Figure 5:**
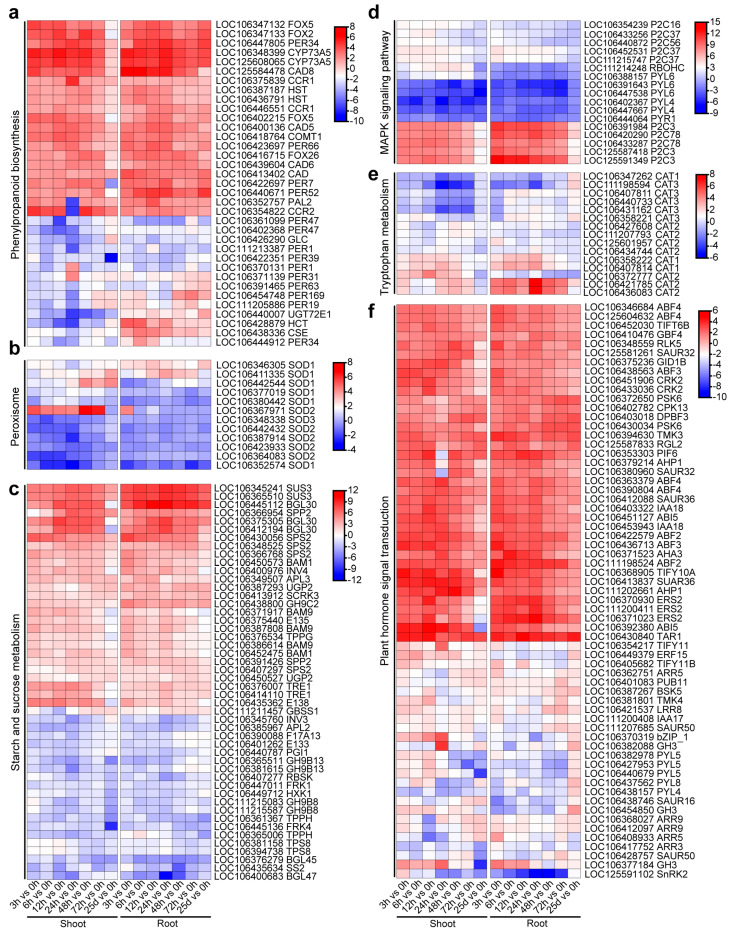
The expression pattern of selected DEGs under salt stress in rapeseed shoots and roots. Heatmap showing the DEGs involved in phenylpropanoid biosynthesis (**a**), tryptophan metabolism (**b**), starch and sucrose metabolism (**c**), MAPK signaling pathway (**d**), peroxisome (**e**) and plant hormone signal transduction (**f**) in shoots and roots under salt stress using log_2_-transformed TPM values. Red and blue colors represent up-regulated and down-regulated genes compared to 0 h, respectively. The heatmap was generated using the TBtools software (version 2.476).

Furthermore, many candidate genes related to phenylalanine biosynthesis, peroxisome, starch and sucrose metabolism, MAPK signaling pathway, tryptophan metabolism and plant hormones were obtained (Figure 5).

A total of 37 DEGs were identified in phenylalanine biosynthesis, the major structural genes directly or indirectly involved in lignin biosynthesis, such as phenylalanine deaminase (PAL), cinnamyl alcohol dehydrogenase (CAD), manganate O-hydroxycinnamoyl transferase (HCT), ferulic acid 5- hydroxylase (F5H), caffeic acid 3-O-methyltransferase (COMT), and cinnamoyl-CoA reductase (CCR) are shown in Figure 5a.

The expression level of *CYP73A5* (*LOC106348399* and *LOC125608065*), *PAL2* (*LOC106352757*), *CCR1* (*LOC106375839*), *CAD1* (*LOC106413402*), *CAD5* (*LOC106400136*), *COMT1* (*LOC106418764*), *PER66* (*LOC106423697*) and *PER34* (*LOC106447805*) were notably increased under salt stress. The abundance of these peroxidase genes decreased, such as *PER47* (*LOC106361099* and *LOC106402368*), *PER1* (*LOC106370131* and *LOC111213387*) and *PER39* (*LOC106422351*), evidently in shoots and roots at different stress stages (Figure 5a, Table S4). Among them, the *HCT* (*LOC106428879*) and *CSE* (*LOC106438336*) exhibited opposite expression patterns across tissues, with suppressed expression in shoots and elevated levels in roots.

The enzyme of superoxide dismutase (*SOD2*, *LOC106367971*) showed opposite expression trends in aboveground and underground tissues (Figure 5b, Table S5). In Figure 5d and Table S8, we found that the catalase genes (*CAT2, LOC106421785* and *LOC106436083*) were more strongly up-regulated in roots than in shoots. Meanwhile, *CAT1* (*LOC106358222* and *LOC106407814*) was mainly up-regulated at the early-term salt stress in both tissues.

After salt stress, genes associated with sucrose synthesis (*SUS3*), sucrose phosphate synthase (*SPS2*), glucose-1-phosphate adenylyltransferase large subunit 3 (*APL3*), sucrose–phosphate phosphatase (*SPP2*), glucose-1-phosphate uridylyltransferase 2 (*UGP2*), beta-glucosidase 30 (*BGL30*) and starch degradation (*BAM1*, *BAM9*) were strongly up-regulated, trehalose–phosphate phosphatase H (*TPPH*), endoglucanase (*GH9B13*, *GH9B8*), beta-glucosidase (*BGL45*, *BGL47*) and fructokinase (*FRK1*, *FRK4*) were down-regulated. Notably, several O-glycosyl hydrolase family genes, including glycosyl hydrolase 9B13 (*LOC106381615*) and endo-1,3-beta-glucosidase 12 (*LOC106390088*), exhibited significant up-regulation in roots under long-term salt stress (Figure 5c, Table S6).

Abscisic acid receptor genes, including *PYR1* (*LOC106444064*), *PYL4* (*LOC106402367* and *LOC106447667*) and *PYL6* (*LOC106388157*, *LOC106391643* and *LOC106447538*), were down-regulated, while the transcription of PP2C family genes was activated, associated with the MAPK signaling pathway in shoots and roots after salt stress (Figure 5d, Table S7). Notably, the expression of respiratory burst oxidase homologs *RBOHC* (*LOC111214248*) was significantly down-regulated in roots compared to the shoots, suggesting a tissue-specific modulation of ROS homeostasis under salt stress.

For plant hormone signal transduction, the bZIP transcription factors ABSCISIC ACID-INSENSITIVE 5-like (*ABF2*, *ABF3* and *ABF4*) and G-BOX BINDING FACTOR 4 (*GBF4*) were markedly induced by salt stress, and the auxin-biosynthesis gene (*TAR1*) and auxin-responsive gene (*SAUR36*) were up-regulated, indicating a critical role of ABA and auxin signaling in activating stress tolerance pathways (Figure 5d, Table S9).

Taken together, these results indicate that salt stress triggers transcriptional reprogramming with distinct tissue-specific and time-dependent patterns involving redox balance, secondary metabolism, carbohydrate homeostasis and multiple signaling pathways, which collectively form a sophisticated regulatory network crucial for plant adaptation to salt stress.

### 3.4. The Weighted Gene Co-Expression Network Construction and Analysis

**Figure 6:**
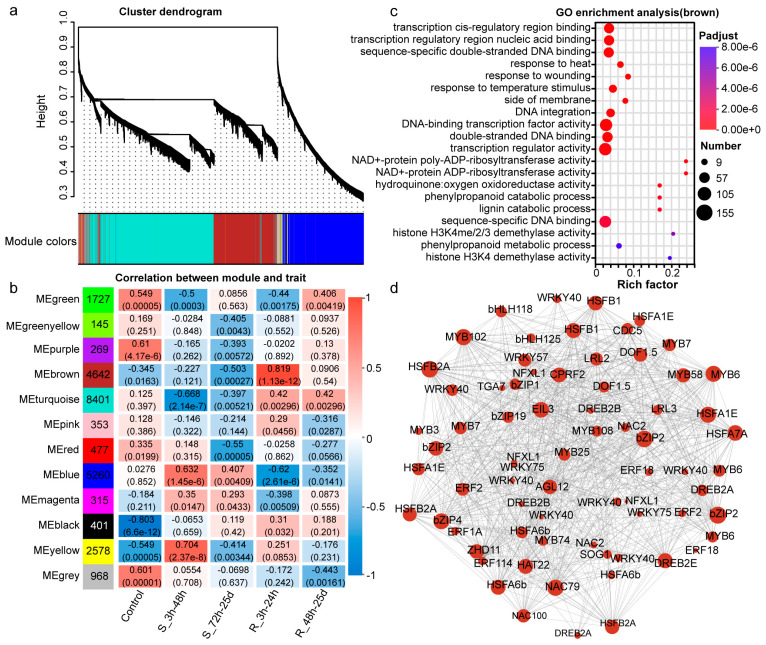
WGCNA-based co-expression network analysis of differentially expressed genes in salt-treated shoot and root tissues. (**a**) Branches constitute the 15 modules labeled in different colors. Black lines and dots represent specific genes assigned to modules. (**b**) Module-trait relationship showing the modules correlated with control and salt stress. S_3 h–48 h and S_72 h–25 d, respectively, refer to early/long-term salt stress in shoots. R_3 h–24 h and R_48 h –25 d, respectively, refer to early/long-term salt stress in roots. Numbers in colored modules denote gene quantities. Values above boxes are Pearson correlation coefficients, and parenthetical figures stand for *p* values. Red indicates positive correlation, blue stands for negative correlation. (**c**) Top 20 GO enrichment of DEGs from MEbrown module. (**d**) Co-expression network constructed by gene correlation within MEbrown module.

Weighted gene co-expression network analysis (WGCNA) with RNA-seq data was performed to screen core genes in different tissues and stress periods. The 25,536 genes were further divided into 12 modules using WGCNA (Figure 6a). Samples were clustered regularly based on control, and the tissue (shoots and roots) and the time after salt stress. In Figure 6a, each module was represented by a different color. Homologous module genes were gathered together, and diverse modules were well differentiated (Figure 6a).

We focused on modules that were negatively correlated with the control and positively correlated with salt stress, including MEbrown, MEmagenta, MEblack, and MEyellow modules (Figure 6b). In co-expression networks, hub genes refer to highly interconnected core genes with prominent topological connectivity (Figure S5, Table S10). In particular, the module MEbrown (r = 0.819, *p* value = 1.13 × 10^−12^) was highly correlated with roots under early-term salt stress.

Related root genes displayed notable up-regulation at early stress phase (Figure 6b and Figure S4a), suggesting that the DEGs are involved in response to salt stress, like Cytokine-induced anti-apoptosis inhibitor (*DRE2*), abscisic acid-insensitive 5 (*ABI5*), scarecrow-like transcription factor (*PAT1*), NAD(H) kinase 1 (*NADK1*), histidine protein methyltransferase 1 homolog (*MET18*), E3 ubiquitin-protein ligase (*RNF5*), ubiquitin-conjugating enzyme E2 34 (*UBC34*), cysteine-rich transmembrane module 13 (*WIH1*), trehalose–phosphate phosphatase G (*TPPG*), cinnamyl alcohol dehydrogenase 5 (*CAD5*), PP2A regulatory subunit (*TAP46*), mitogen-activated protein kinase 6 (*MPK6*) and lifeguard 2 (*LFG2*) (Figure S5a).

Among these, the MEmagenta module was positively correlated in shoots under salt stress (Figure 6b and Figure S4b), which involved in circadian rhythm, light and abiotic stimulus, such as potassium transporter (*BNAC08G27250D*), protein REVEILLE 4 (*RVE4*), nuclear transcription factor Y subunit A-4 (*NFYA4*), solanesyl diphosphate synthase 1 (*SPS1*), dehydrogenase/reductase SDR family member 12 (*DHRS12*), tyrosine-specific transport system isoform X1 (*TRYP)*, RNA polymerase sigma factor (*SIGE*), dehydrogenase/reductase SDR family member 12 (*DHRS12*), glutathione S-transferase U18 (*GSTU18*), quinone-oxidoreductase homolog (*CEQORH*), ABC transporter F family member 5 (*ABCF5*), carotenoid cleavage dioxygenase 1 (*CCD1*), B-box zinc finger protein 32 (*BBX32*) and aldehyde dehydrogenase family 3 member I1 (*ALDH3I1*) (Figure S5b).

Whereas the hub genes in MEblack module mainly involved in energy and metabolic pathways, contributing to plant salt stress tolerance, which showed a positive correlation in roots under salt stress (Figure 6b and Figure S4c), including Maternal effect embryo arrest 47 (*MEE47*), F-BOX protein (*FB262*), sterile alpha motif (SAM) domain-containing protein (*BNAC01G39700D*), BTB/POZ domain-containing protein (*PRL1*), transposase (*TRA1*), nijmegen breakage syndrome 1 (*NBS1)* and a proline-rich protein SICKLE (*SIC*) (Figure S5c).

In addition, the MEyellow module exhibited a strong positive correlation with both shoots and roots at the early-term of salt stress (Figure 6b and Figure S4d), which hub genes involved oxygen-containing compound and plant hormone signal transduction, such as Zinc finger A20 and AN1 domain-containing stress-associated protein 9 (*SAP9*), glycine-rich RNA-binding protein GRP2A (*RBG7*), F-box protein (*SKP1*), alpha-aminoadipic semialdehyde synthase (*LKR*/*SDH*), persulfide dioxygenase ETHE1 homolog (*GLY3*), PLATZ transcription factor family protein (*PLATZ7*), Ras-related protein (*RABC2B*), mitogen-activated protein kinase kinase kinase 3 (*MAPKKK17*), aspartate aminotransferase 3 (*ASP3*), homeobox–leucine zipper protein (*ATHB-7*), basic leucine zipper 1 (*bZIP1*), ABI5 binding protein 3 (*AFP3*), vacuolar amino acid transporter 1 (*AVT1)* and NAC domain-containing protein 19 (*NAC019*) (Figure S5d). Collectively, these co-expression modules and hub genes regulate the salt stress response of rapeseed in a tissue- and time-specific manner.

Furthermore, GO enrichment results revealed that MEbrown module genes functioned in DNA-binding transcription factor, transcription regulator and *cis*-regulatory region binding under salt stress (Figure 6c). KEGG enrichment analysis also showed that most of these DEGs are related to basal transcription factors and plant hormone signal transduction pathways (Figure S6). Genes in the MEbrown module participate in basal transcription factor functions and plant hormone signaling pathways, regulating plant responses to salt stress.

In above pathways, several transcription factors, mainly including WRKY (*WRKY40*, *WRKY57* and *WRKY75*), NAC (*NAC2*, *NAC79* and *NAC100*), MYB (*MYB3*, *MYB6*, *MYB7*, *MYB58*, *MYB74*, *MYB102* and *MYB108*), bZIP (*bZIP1*, *bZIP2*, *bZIP4* and *bZIP19*), DREB (*DREB2A*, *DREB2B* and *DREB2E*) and bHLH (*bHLH118* and *bHLH125*) were also found in the regulatory network under salt stress (Figure 6d). These results suggest that the MEbrown module, along with its associated transcription factors, may contribute to salt stress tolerance in rapeseed primarily by modulating transcriptional processes and plant hormone signal transduction pathways.

qRT-PCR analysis was performed to validate the quality of the RNA-Seq data. Nine genes were randomly selected for qRT-PCR analysis. The expression profiles of these genes, as revealed by qRT-PCR, were similar to those observed in the RNA-Seq data. The correlation coefficient (R) between the RNA-Seq and qRT-PCR analyses was ≥ 0.77 for all genes tested (Figure S8), indicating the reliability of our transcriptomic profiling data.

## 4. Discussion

### 4.1. Physiological Effects of Antioxidant Enzymes in Rapeseed Aerial and Root Tissues Under Salt Stress

As a major oil-bearing crop, *B. napus* derives from the interspecific hybridization and chromosome doubling of diploids ancestral species [27]. As the primary organs exposed to saline environments, roots perceive salt stress earlier and are likely to suffer more severe damage than aboveground tissues [28]. Wang et al. [19] performed root transcriptome analysis of rapeseed and mainly explored salt-responsive transduction and scavenging of ROS under salt stress. Roots are the initial tissue exposed to salinity, and exploring their physiological alterations matters greatly, along with tissue-specific differences between shoots and roots, is crucial for elucidating salt stress tolerance mechanisms.

As such, salt-tolerant rapeseed serves as an ideal research system, with both shoots and roots providing sensitive biological targets for studying the molecular mechanisms underlying plant salt tolerance and adaptation. In addition, the use of a single genotype restricts the generalisability of our conclusions.

Nevertheless, few studies have systematically compared the divergent responses of different plant tissues to early- and long-term salt stress. In this study, the biomass of shoots and roots was severely decreased under 275 mM NaCl salt treatment (Figure 1). Previous studies have adopted 200–250 mM NaCl for salt treatment during the germination stage [29,30]. Low salt concentrations failed to produce significant genotypic differences between different genotypes [31].

It has been reported that Huayouza 62 still kept a high germination rate under 200 mM NaCl [32]. This concentration is consistent with that of Ma et al. [31], who reported that 272 mM NaCl was the threshold to exhibit a significant decreasing trend. Thus, 275 mM NaCl was applied as the high salt treatment concentration in the present experiment. After 25 days of salt stress, the growth of salt-tolerant rapeseed was restrained and its biomass declined (Figure 1).

Abiotic stress triggers massive ROS accumulation in plants. Meanwhile, plants activate SOD, CAT and POD antioxidant enzymes to mitigate ROS-induced damage [33]. In this study, SOD activity in shoots and roots rose continuously, presenting notable elevation at 24 h and 48 h relative to their controls under salt stress (Figure 2a,b). Root tissue exhibited low SOD activity and less ROS scavenging ability than shoots. Salinity sharply raised POD activity in both tissues, with roots showing higher POD levels (Figure 2c,d).

Saline treatment triggered obvious increases in POD and CAT activity yet decreased SOD levels in wheat leaves and roots [34]. Under long-term salt treatments, the *BnBBX22.A07*-OE lines had a significantly lower MDA content compared with the control rapeseed [11]. Shoot CAT activity displayed a further decline, observed with prolonged exposure to NaCl treatment (Figure 2e,f). Likewise, *CAT2* (*LOC106421785* and *LOC106436083*) dropped in early-term shoots. Another, *CAT2* (*LOC106372777*), was up-regulated in shoots and down-regulated in roots (Figure 5e). This indicates divergent temporal and tissue-specific expression of *CAT2* homologs in rapeseed.

Different copies show distinct expression patterns, with opposite regulatory trends in shoots and roots. This imbalance partially disrupts ROS scavenging capacity and breaks antioxidant homeostasis across plant tissues. Thus, rapeseed roots exhibited higher MDA content than shoots, especially under long-term salt treatment (Figure 2g,h). MDA accumulated continuously as salt stress persisted, implying that the antioxidant system gradually reached saturation and suffered partial functional impairment. This is also consistent with Song et al. [35] and our observations of excessive Na^+^ accumulation and significant biomass reduction under salt stress.

This direct and prolonged high-salt treatment can well mimic the abrupt rise in soil salinity triggered by frequent seawater intrusion in coastal tidal flat areas of Shanghai. Therefore, we mainly focused on exploring the long-term adaptive salt tolerance mechanisms of rapeseed under sustained severe salt stress conditions. Nevertheless, this experimental setup still cannot fully represent the overall dynamics of natural soil salinization. Our research is oriented toward the adaptation and improvement of coastal saline–alkali land.

Several oxidases are responsible for ROS production in the apoplast, such as Class III peroxidases (CIII PRXs), apoplastic peroxidase (POD) and cell wall-associated peroxidase (POX). In our study, several key flavonoid biosynthesis genes, such as *HST*, *CAD* and multiple *PER* family members were significantly up-regulated, with *PER34* (*LOC106444912*) specifically induced at early-term, and *PER31*, *PER63* and *PER169* exclusively up-regulated in roots under long-term salt stress (Figure 5a).

Taken together, our findings highlight that the orchestrated induction of phenylpropanoid and flavonoid pathway genes in roots, especially in a stage-specific manner, contributes substantially to fighting against salt stress. Flavonoids scavenge ROS to relieve oxidative damage and improve salt tolerance. Multiple functional genes are activated to control ROS levels and protect cells [36].

In this study, a hub gene encoding glutathione S transferases 18 (*GSTU18*), associated with oxidative metabolism, as depicted in Figure S5b, in the MEmagenta module, was positively correlated in shoots at early- and long-term salt stress treatment (Figure 6b). And *UGT72E1*, a UDP-glucosyltransferase, exhibited opposite expression patterns in shoots and roots; it rose in shoots yet fell in roots (Figure 5a). Lower antioxidant activity weakened ROS clearance, reflecting poorer salt tolerance of roots.

### 4.2. Phenylpropanoid Pathway Contributes to Rapeseed Salt Tolerance

Phenylpropanoids facilitate plant growth and stress adaptation, protecting cells from oxidative harm [37]. Starting from phenylalanine, the pathway produces lignin, flavonoids and other defensive metabolites. In this study, salt stress induced up-regulation of *PAL2* (*LOC106352757*) in both shoots and roots, with the exception of a transient down-regulation at 24 h in shoots (Figure 5a). In contrast, *HCT* (*LOC106428879*) was up-regulated in roots but down-regulated in shoots under salt stress (Figure 5a).

*SlCOMT1* overexpression elevates melatonin and proline, lowers ROS, and strengthens tomato salt tolerance [38,39]. Among DEGs, *COMT1* (*LOC106418764*), *CCR1* (*LOC106446551*) and *CCR2* (*LOC106354822*, except for 24 h in shoots), closely associated with most structural genes and metabolites, were found to be up-regulated in shoots and roots under salt stress (Figure 5a). Consistent with prior reports, salt stress boosts *COMT* and *CCR* expression in this study. Heterologous *COMT* expression enhances salt tolerance in *Arabidopsis* via improved osmotic adjustment and antioxidant ability [40,41]. Besides catalytic function, *COMT* also regulates root early-term salt response and oxidative protection (Figure 5a, Table S3).

### 4.3. Phytohormone Signaling and Its Regulatory Functions in Salt Stress Tolerance

Plant hormones fall into two categories. ABA, cytokinin (CK), ethylene, strigolactones (SL) auxin and jasmonic acid (JA) mediate stress responses, while others mainly regulate growth [42]. Over 80 hormone-related DEGs (Figure 5 and Figure S5) potentially modulate rapeseed salt tolerance.

The ABA signaling cascade starts with its recognition by ABA-binding PYR/PYL/RCAR receptors, as it inhibits PP2Cs and activates SnRK2s, triggering downstream ABA-responsive genes [43]. We identified 11 DEGs involved in ABA receptor (*PYL*/*PYR*) that were down-regulated in rapeseed, thereby activating 10 PP2C-related DEGs (Figure 5d,f).

Multiple factors bind SOS2 and suppress its activity to block the SOS pathway without salt stress [44]. In this study, we found that *ABI5* (*LOC106451127*) and *GID1B* (*LOC106375236*) were up-regulated (Figure 5f). DELLA mutant roots resist salt growth inhibition yet are prone to salt-induced death, balancing growth and survival under salinity [45]. In this study, *RGL2* (*LOC125587833*) was significantly up-regulated under salt stress (Figure 5f). Such up-regulation may contribute to a strong antioxidant defense mechanism and promote stress survival in rapeseed.

Overexpressing rice *OsERF922* elevates the shoot Na^+^/K^+^ ratio and weakens salt tolerance [46]. In this study, salt stress caused greater Na^+^ accumulation in shoots than roots, increasing the Na^+^/K^+^ ratio by 46.26% and 26.33% in shoots and roots, respectively (Figure 1d–f). The expression profiles of key ion transporters, including the sodium/hydrogen exchanger (*NHX*) family (including salt overly sensitive 1 (*SOS1*), salt overly sensitive 3 (*SOS3*), and components of the potassium transport system such as Arabidopsis K^+^ channel (*ATK*), high-affinity K^+^ transporter (*HAK*), stelar K^+^ outward rectifier (*SKOR*), K^+^ channel outward rectifier (*KCO*) and high-affinity K^+^ transporter 1 (*HKT1*), were presented in Figure S7a–e and Tables S11–S15. These genes also exhibited marked tissue-specific spatiotemporal differential expression. Consistently, the transcript levels of *NHX2*, *NHX6*, *SOS3* and *HKT1* (*LOC106358873* and *LOC106443963* in 25 days, and *LOC106376304*) were markedly up-regulated in roots but down-regulated in shoots (Figure S7a–e).

This evidence demonstrates that roots maintain stronger Na^+^ exclusion capacity and a more robust potassium transport system to cope with prolonged salt stress, which helps roots better adapt to a saline environment. This was accompanied by down-regulated *ERF15* (*LOC106449379*), particularly in roots (Figure 5f). The ethylene receptors ethylene response (*ETR1*/*2*), ethylene response sensor (*ERS1*/*2*) and ethylene insensitive 4 (*EIN4*) are active in the absence of ethylene.

In our study, *ERS2* (*LOC106370930*, *LOC111200411*, and *LOC106371023*) showed significant up-regulation, especially in the early-term salt stress in roots (Figure 5f). Ethylene signaling enhances rapeseed salt tolerance. Salt stress activates the SL transduction pathway by up-regulating the expression of its key components *CCD1* (*LOC106444915*), which was positively correlated with shoot development under salt stress and assigned to the MEmagenta module (Figure 6c and Figure S5b).

And, we identified significant down-regulation of *ARR3* (*LOC106417752*), *ARR5* (*LOC106408933*), and *ARR9* (*LOC106368027* and *LOC106412097*) in both shoots and roots of rapeseed under salt stress (Figure 5f). ARR1 and ARR12 can repress the transcription of *HKT1*. This gene encodes a high-affinity potassium transporter that mediates root Na^+^ recycling from xylem sap and subsequent vacuolar sequestration [47].

Cytokinin binding triggers AHK autophosphorylation, followed by phosphate transfer to AHPs [48]. In this study, two AHP homologs (*LOC106379214* and *LOC111202661*) were significantly up-regulated in rapeseed under salt stress, indicating enhanced CK signaling transduction (Figure 5f). Together, ABA-suppressed CK signaling benefits rapeseed survival under salt stress.

### 4.4. Regulation of Starch and Sucrose Metabolism in Response to Salt Stress

Sugars sense stress, relay signals and regulate genes for osmotic balance, ROS clearance and energy supply [49]. As shown in Figure 4, for KEGG starch and sucrose metabolism DEGs under salt stress, the RF value in shoots was 0.099 (72 genes) at the early-term and 0.095 (69 genes) at the long-term. In roots, the RF value was 0.152 (110 genes) at the early-term and 0.102 (74 genes) at the long-term. Starch and sucrose metabolism respond more strongly to early-term salt stress. This observation is consistent with the findings of Chen et al. [37]. Moderate DEG enrichment in the early stage indicates metabolic pathways undergo adaptive changes responding to short-term environmental variation.

SUS, SPS and INV families dominate sugar metabolism, showing tissue-specific expression variations under salt stress [50]. In our study, we observed that up-regulation of *SUS3* (*LOC106345241* and *LOC106365510*), *SPS2* (*LOC106430056*, *LOC106348525*, *LOC106366768* and *LOC106407297*) and sucrose breakdown gene *INV4* (*LOC106400976*) coupled with down-regulation of *INV3* (*LOC106345760*) and *SS2* (*LOC106435634*) (Figure 5c, Table S6), which may contribute to the regulation of sucrose level in rapeseed under salt stress.

ABA-activated SnRK2s modulate osmotic balance via the AREB/ABF-SnRK2 pathway by inducing *BAM1* and *AMY3* to produce osmotic substances [51]. In this study, *BAM1* (*LOC106450573* and *LOC106452475*) and *BAM9* (*LOC106371917*, *LOC106386614* and *LOC106387808*) were strongly up-regulated under salt stress, consistent with enhanced osmotic adjustment (Figure 5c).

Notably, one *SnRK2* homolog (*LOC125591102*) was significantly down-regulated, whereas key abscisic acid-responsive element-binding factor ABF2/3/4 in rapeseed were transcriptionally induced (Figure 5d). These observations suggest that the up-regulation of *BAM* genes may rely on other functional SnRK2 isoforms, or on ABF activation through alternative signaling cascades independent of the down-regulated *SnRK2*. Partial *BAM1* activation in the triple mutant probably results from functional ABF1 involved in leaf osmotic stress signaling [52]. These findings reveal transcriptional patterns of rapeseed salt responses and support subsequent relevant research.

### 4.5. MAPK Cascade Signaling in Response to Salt Stress

MPK cascades widely exist across diverse organisms, participating in plant ionic, osmotic and oxidative stress responses [53]. MPK8 links phosphorylation, calcium and ROS signals, while AtMPK6, activated by phosphatidic acid, targets SOS1 downstream [54,55].

The transcriptional factor OsWRKY53 has been identified and demonstrated to act as a negative modulator of salt tolerance, and directly trans-regulates the expression of OsMKK10.2 in promoting shoot Na^+^ exclusion [56]. Several core genes were activated at initial salt stress. *GmMPK6* boosts soybean salt resistance by regulating *GmRbohI1* to promote ROS production [57].

In our study, *MAPK6* (*LOC106388342*) exhibited a positive correlation with roots under early-term salt stress and was clustered in the MEbrown module (Figure S5a), while *MAPKKK17* was positively associated with both shoots and roots at the early-term salt stress and grouped into the yellow module (Figure S5d).

### 4.6. Transcription Factor-Mediated Regulatory Networks in Salt Stress Responses

Transcription factors regulate plant growth and stress responses by binding gene promoter cis-elements [58]. Major functional families include MYB, NAC, ERF, bZIP, bHLH and WRKY. We employed WGCNA to construct a co-expression network of genes that are highly correlated with different time and tissues of salt stress and identified key hub genes (Figure 6). Consistent with the previous studies that TFs could positively or negatively participate in salt tolerance, we also observed that WRKY (*WRKY40/57/75*), NAC (*NAC2/79/100*), MYB (*MYB3/6/7/58/74/102/108*), bZIP (*bZIP1/2/4/19*), DREB (*DREB2A/2B/2E*) and bHLH (*bHLH118/125*) detected significantly induced in response to 3–24 h (ealy-term) salt treatment in roots (Figure 6d). PIFs belong to the bHLH transcription factor family and suppress plant photomorphogenesis [59,60]. Moreover, PIF4 also lowers salt tolerance via modulating stress-related gene expression [61].

In our study, we identified significant up-regulation of *PIF6* (*LOC106353303*) and DELLA protein *RGL2* (*LOC125587833*) under salt stress in shoots and roots (Figure 5f). These findings suggest a close link between light and salt stress signaling. Notably, the sustained elevation of MDA content at both early- and long-term salt stress may also be exacerbated by Fenton reaction-mediated iron-dependent oxidative damage. *Ferritin* (*Fer*) genes are critical regulators of plant oxidative stress resistance, functioning primarily by sequestering excess free ferrous ions (Fe^2+^) in a non-toxic form, thereby preventing iron-induced oxidative toxicity and playing an essential role in salt stress tolerance [62].

In this study, two *ferritin1* and *ferritin2* isoforms (*LOC106427233*/*LOC106433816* and *LOC106345571*) showed only limited up-regulation with relatively low transcript abundance under salt stress. This insufficient induction of ferritin genes failed to effectively chelate the excess free Fe^2+^ released under oxidative stress, further aggravating lipid peroxidation and cell membrane damage.

**Figure 7:**
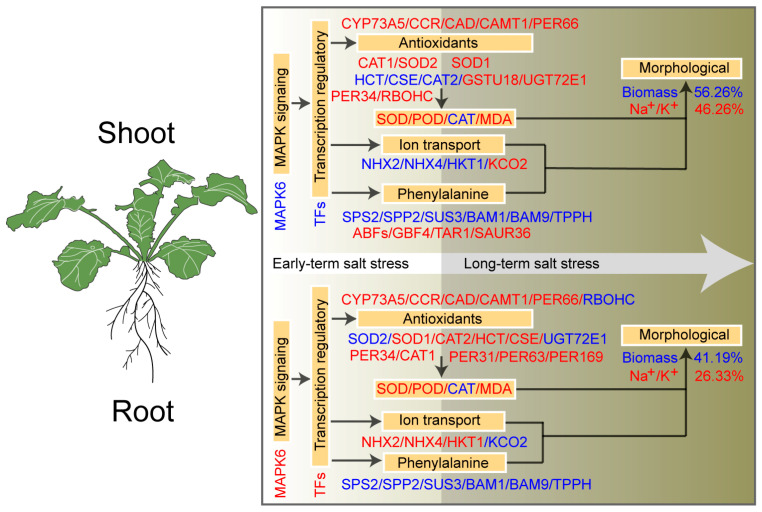
A schematic model of tissue- and time-specific salt stress responses in *B. napus*. Light and dark regions represent early- and long-term salt stress, respectively. Red and blue texts indicate up- and down-regulated DEGs, arrows show the stress time course of shoots and roots. Blue biomass indicates decreased levels compred with their CK, red Na^+^/K^+^ represent increased levels.

Collectively, we propose a preliminary regulatory model summarizing the tissue- and time-specific responses of *B. napus* to salt stress (Figure 7). As illustrated, shoot and root tissues adopt distinct adaptive strategies under both early- and long-term salt stress, where MAPK signaling and transcription factors act as upstream regulators to coordinate the activation of antioxidant systems, phenylpropanoid metabolism, starch and sucrose pathways, and plant hormone signaling.

These divergent regulatory patterns are directly consistent with our physiological data, where significant differences in SOD, POD, CAT, and MDA responses between shoots and roots. While both tissues activate antioxidant pathways to mitigate oxidative damage, differences in sugar metabolism and hormone signaling likely contribute to the tissue-specific impacts on biomass accumulation and Na^+^/K^+^ homeostasis. This integrated model connects the identified hub genes, key pathways, and physiological phenotypes, providing a coherent framework to understand the mechanisms underlying tissue- and time-specific salt tolerance in *B. napus*.

## 5. Conclusions

This study conducted transcriptome analysis and physiological detection to suggest the salt tolerance mechanisms of *B. napus* shoots and roots under early- and long-term stress. Multiple pathways, including peroxisome and tryptophan metabolism, plant hormone transduction, phenylpropanoids and sugars, jointly regulate antioxidant defense and osmotic balance to cope with salt damage.

Na^+^/K^+^ ratio, antioxidant responses and expression profile displayed distinct tissue specificity. Transcriptome samples were well divided by stress duration, and hub genes identified showed strong relevance to early-term salt response in root tissues. The results enrich our knowledge of salt adaptive mechanisms in rapeseed, and provide valuable molecular candidates for breeding salt-tolerant cultivars.

## Data Availability

The data that support the findings of this study are openly available in [the Sequence Read Archive (SRA) database of National Center for Biotechnology Information (NCBI)] at [https://www.ncbi.nlm.nih.gov/bioproject/PRJNA1449748/ (accessed on 6 April 2026)], reference number [PRJNA1449748].

## Supplementary Materials

The following supporting information can be downloaded at: https://www.mdpi.com/article/10.3390/antiox15060708/s1, Table S1: statistics and evaluation of transcription sequencing data of each sample; Table S2: alignment rate between the RNA-seq data and the reference transcriptome; Table S3: primers in RT-qPCR; Table S4: differentially expressed genes enriched in the phenylpropanoid biosynthesis; Table S5: differentially expressed genes enriched in the peroxisome; Table S6: differentially expressed genes enriched in the starch and sucrose metabolism; Table S7: differentially expressed genes enriched in the MAPK signaling pathway; Table S8: differentially expressed genes enriched in the tryptophan metabolism; Table S9: differentially expressed genes enriched in the plant hormone signal transduction; Table S10: hub genes in the MEbrown, MEmagenta, MEblack and MEyellow module; Table S11: expression level of NHX family genes; Table S12: expression level of *HKT1* gene; Table S13: expression level of *HAK* family genes; Table S14: expression level of *SOS3* genes; Table S15: expression level of potassium channel genes; Figure S1: multivariate statistics analysis of transcriptome data of each sample under salt stress in rapeseed shoots and roots; Figure S2: GO enrichment analysis of transcriptome data of each sample under salt stress in rapeseed shoots and roots; Figure S3: enrichment of GO term from the ‘biological process’ category in genes; Figure S4: standardized TPM expression profiles for MEbrown, MEmagenta, MEblack and MEyellow module genes; Figure S5: gene co-expression networks with the greatest hubness in the MEbrown, MEmagenta, MEblack and MEyellow module; Figure S6: gene expression characteristics of the MEbrown module; Figure S7: expression profiles of ion transporter genes; Figure S8: Correlation between expression profiles of selected genes obtained from RNA-seq and RT-qPCR analysis.

## Abbreviations

The following abbreviations are used in this manuscript:

ROS	Reactive oxygen species
SOD	Superoxide dismutase
POD	Peroxidase
CAT	Catalase
MDA	Malondialdehyde
WGCNA	Weighted gene co-expression network analysis
GO	Gene ontology
KEGG	Kyoto encyclopedia of genes and genomes
RF	Rich factor
TFs	Transcription factors
