# Supplementary material for: Transcriptomic Analysis Suggests Shoots and Roots-Specific Antioxidant Responses to Early-/Long-Term Salt Stress in Brassica napus
Source: Antioxidants (Basel). 2026 Jun 3;15(6):708. doi: 10.3390/antiox15060708 (PMC13295495; doi:10.3390/antiox15060708)
Supplement: Supplementary file 1 [file antioxidants-15-00708-s001.zip › antioxidants-4294634-supplementary/Figures S1 to S8.pdf]

Supplementary file

Transcriptomic Analysis Suggests Shoots and Roots-Specific Antioxidant Responses to Early-/Long-Term Salt Stress in *Brassica napus*

Xianmin Meng, Lei Lei, Weirong Wang, Hongwei Li and Jifeng Zhu *

Key Laboratory of Germplasm Innovation and Genetic Improvement of Grain and Oil Crops (Co-Construction by Ministry and Province), Ministry of Agriculture and Rural Affairs, Key Laboratory of Agricultural Genetics and Breeding of Shanghai, Crop Breeding and Cultivation Research Institute, Shanghai Academy of Agricultural Sciences, Shanghai 201403, China; mengxianmin@saas.sh.cn (X.M.); leilei@saas.sh.cn (L.L.); 20150230@saas.sh.cn (W.W.); lihongwei@saas.sh.cn (H.L.)

* Correspondence: zhujifeng@saas.sh.cn; Tel.: +86-21-6220-8131

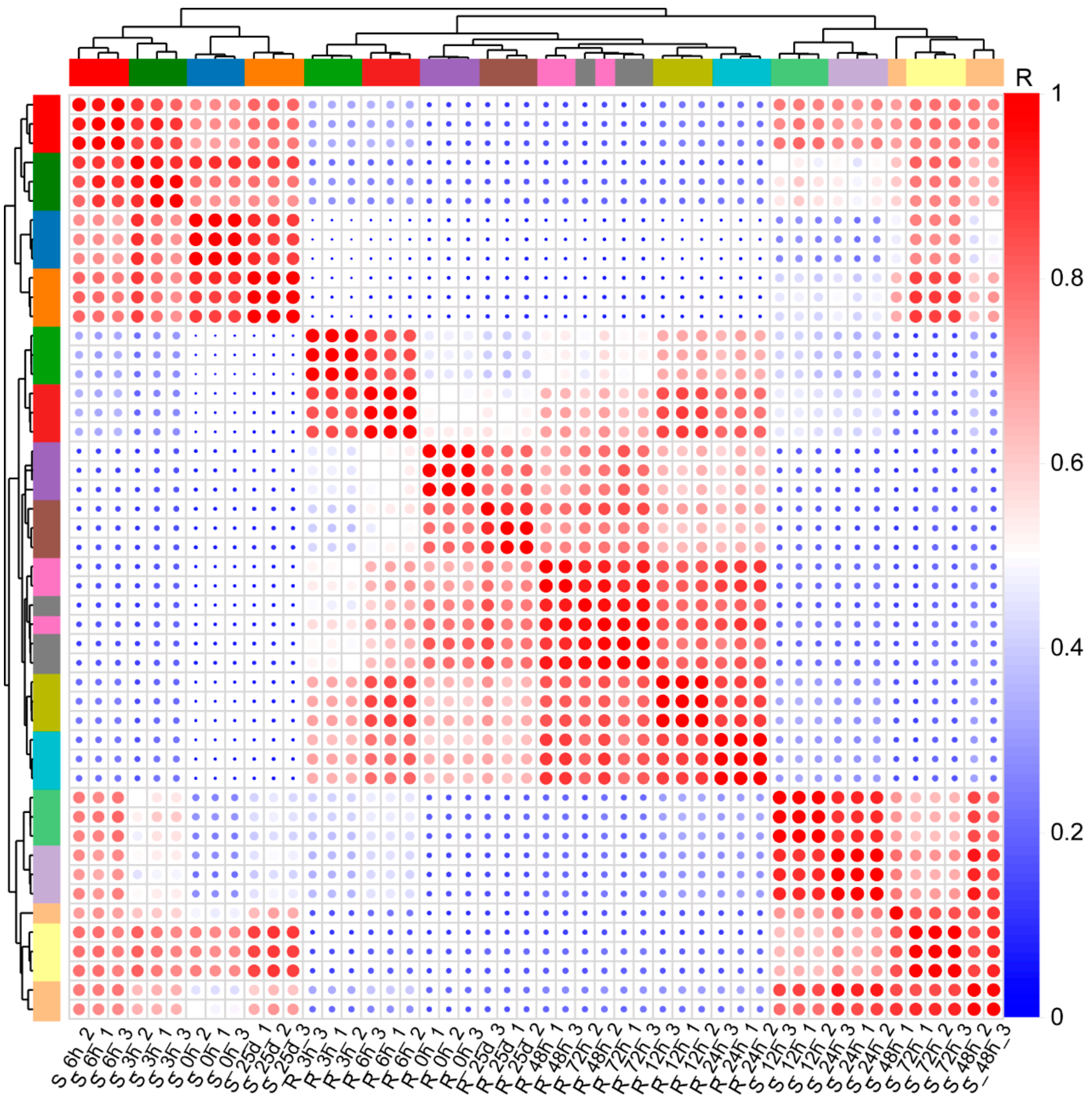

Figure S1. Multivariate statistics analysis of transcriptome data of each sample under salt stress in rapeseed shoots and roots. The Pearson correlation coefficient (R) between samples was calculated and visualized by cluster analysis.

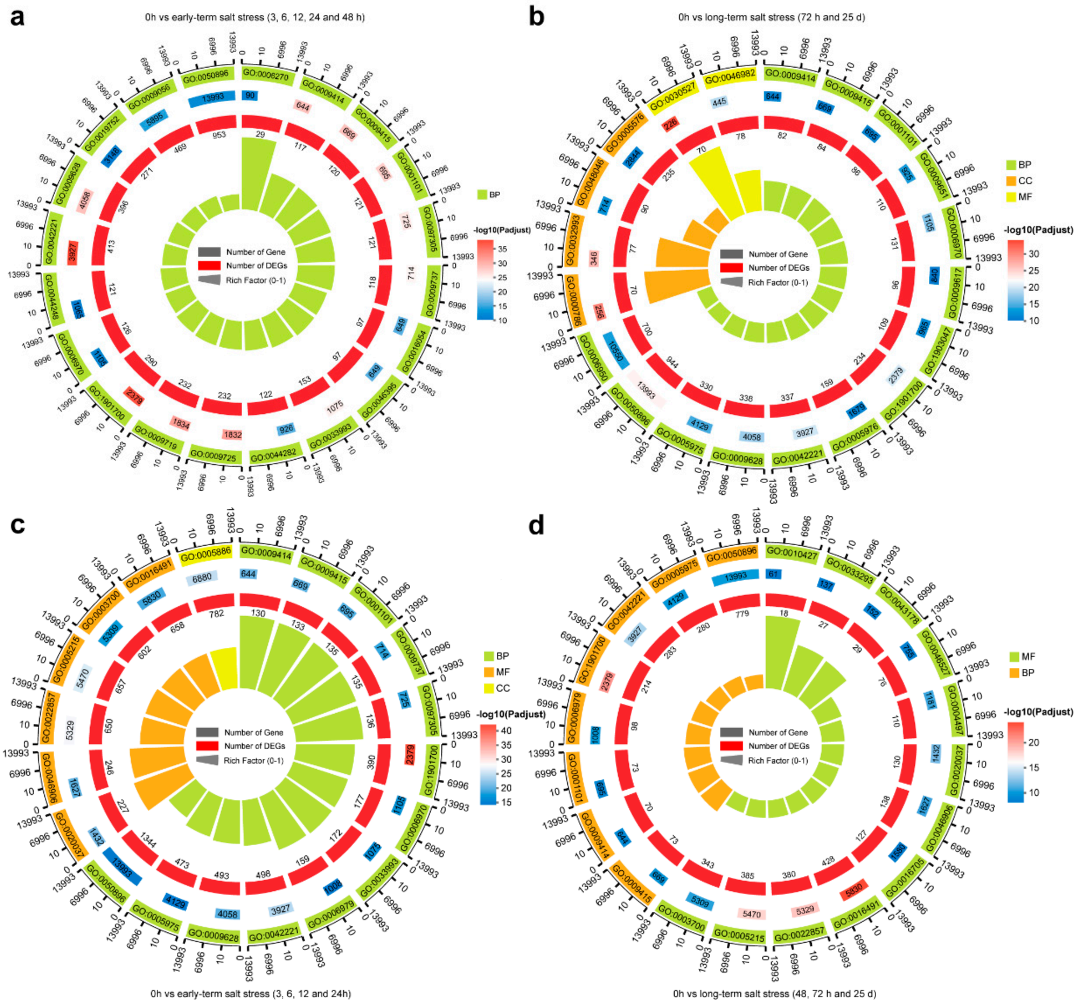

Figure S2. GO enrichment analysis of transcriptome data of each sample under salt stress in rapeseed shoots and roots. **(a-b)** GO enrichment analyses of early- (3, 6, 12, 24 and 48 h) **(a)** and long-term (72 h and 25 d) **(b)** salt treatment in shoots compared to 0 h, respectively. **(c-d)** GO enrichment analyses of early- (3, 6, 12, 24 h) **(c)** and long-term (48, 72 h and 25 d) **(d)** salt treatment in roots compared to 0 h, respectively. The first circle shows the top 20 enriched GO terms, and different colors represent different types of Ontology. The second circle shows the number and *P* value of the GO term in the differential gene background. BP: Biological process; MF: Molecular function; CC: Cellular component. A longer bar indicates a higher number of DEGs backgrounds, and a redder color indicates a lower *P* value. The third circle shows the number of genes in this GO term. The fourth circle shows the rich factor values for each GO term.

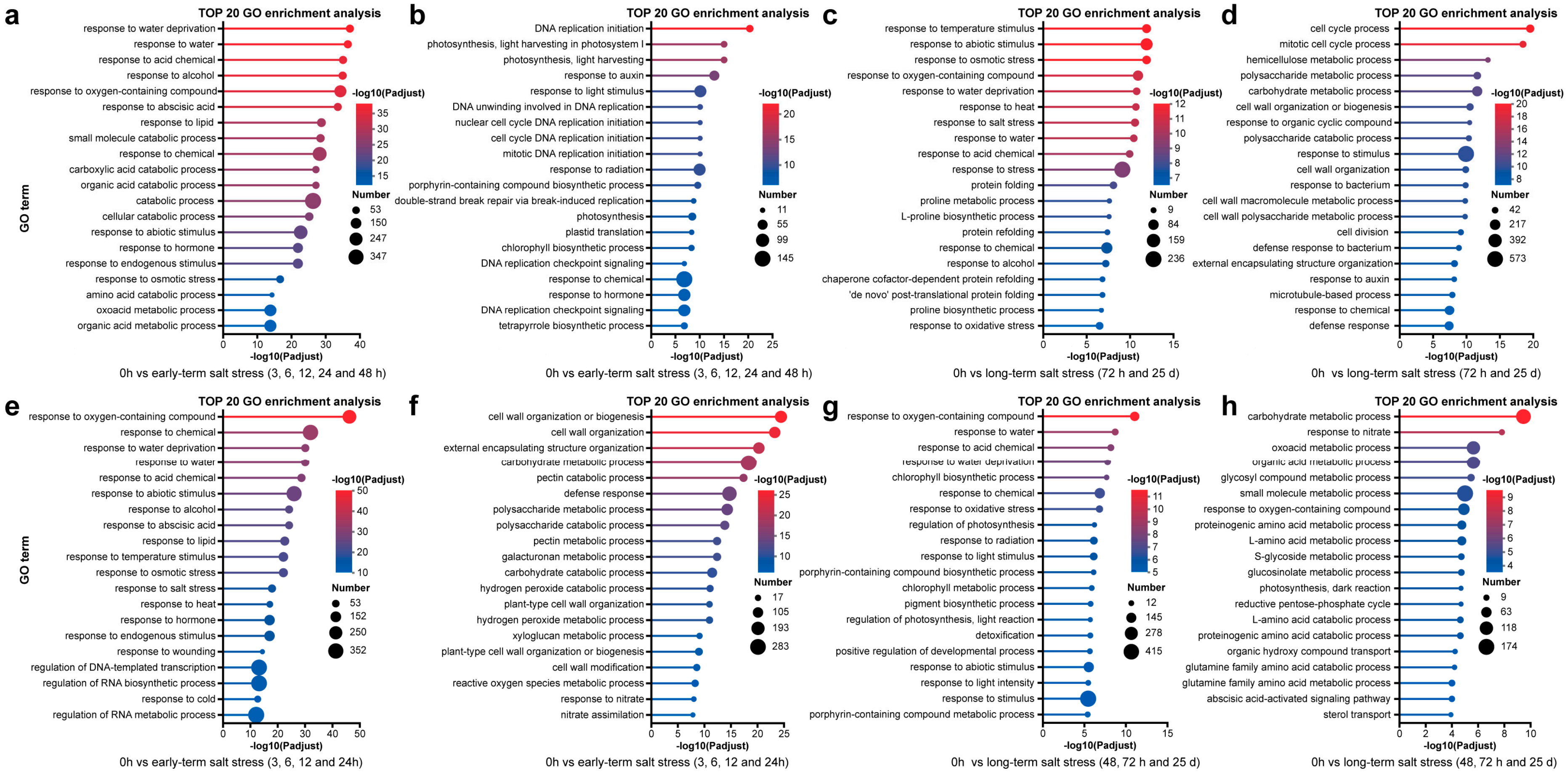

Figure S3. Enrichment of GO term from the 'biological process' category in genes. **(a-d)** GO enrichment of genes with up and down expression trends for early- (3, 6, 12, 24 and 48 h) **(a, b)** and long-term (72 h and 25 d) **(c, d)** salt treatment in shoots compared to 0 h, respectively. **(e-h)** GO enrichment of genes with up and down expression trends for early- (3, 6, 12 and 24 h) **(e, f)** and long-term (48, 72 h and 25 d) **(g, h)** salt treatment in roots compared to 0 h, respectively. The top 20 GO terms with the lowest Qvalues were used to plot the graphs. The vertical axis represents GO terms and the horizontal axis indicates the significance level of enrichment. The size of dots represents the number of genes and the color of dots represents the *P*-adjust.

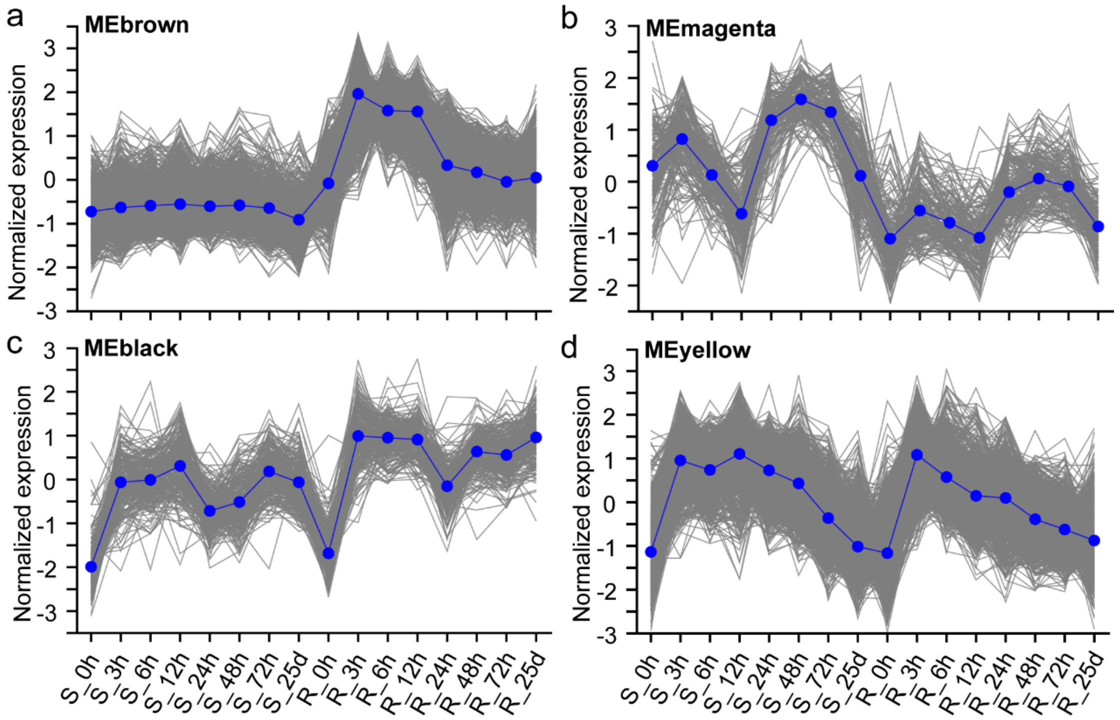

Figure S4. Standardized TPM expression profiles for MEbrown (**a**), MEmagenta (**b**), MEblack (**c**) and MEyellow (**d**) module genes. To better reflect the expression patterns of genes in different samples, z-scores were used to standardize TPM. In the samples, S and R respectively refer to the shoots and roots, and the numbers represent the hours after salt stress treatment. The blue line represents the average of the standardized TPM in each module.

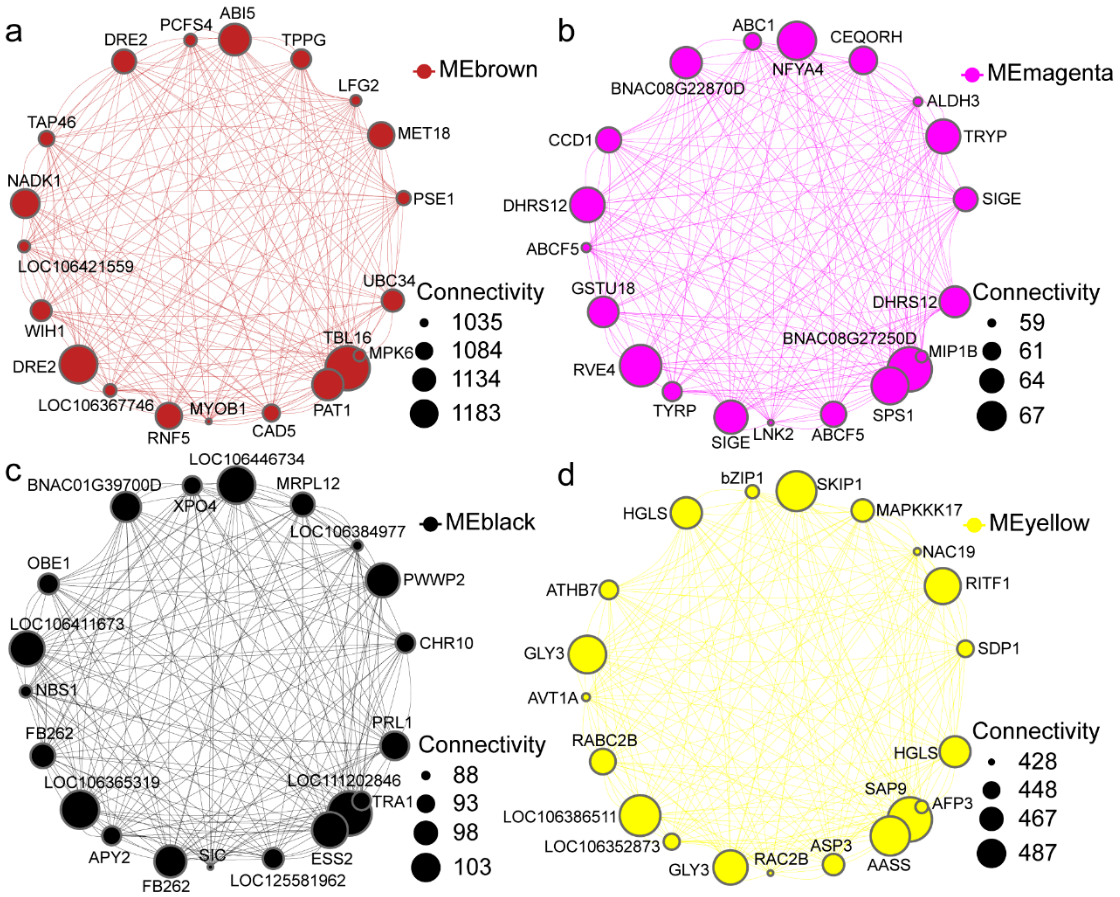

Figure S5. Gene co-expression networks with the greatest hubness in the MEbrown (**a**), MEmagenta (**b**), MEblack (**c**) and MEyellow (**d**) module. The top 20 nodes ranked by 'degree', which were calculated by CytoHubba, were selected as hub nodes. Nodes are represented by circles filled with the module colors. The higher the module connectivity, the higher the 'degree' of the nodes.

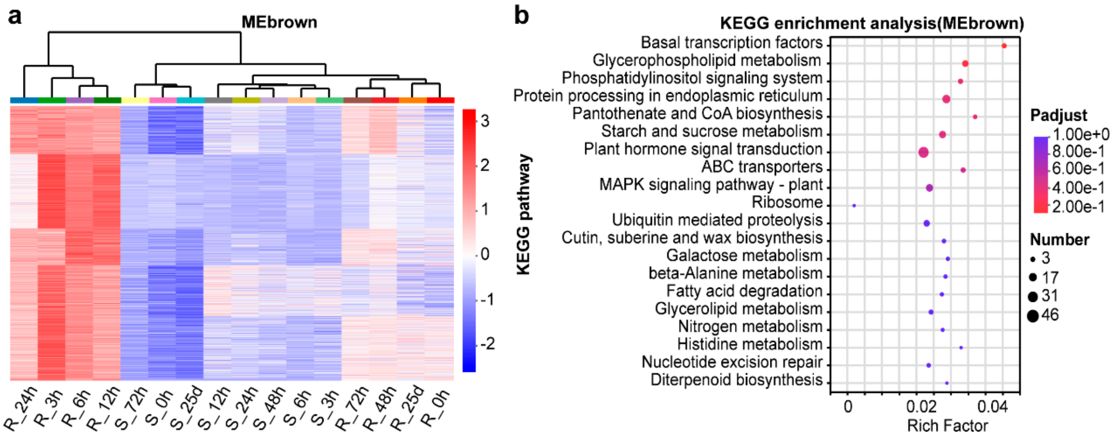

Figure S6. Gene expression characteristics of the MEbrown module. **(a)** Heatmap shows the expression patterns of the co-expressed genes in MEbrown module. Red and blue colors represent the high and low gene expression levels, respectively. The dendrogram above shows the clustering of samples. The closer two sample branches are, the more similar the overall expression patterns between these two samples. **(b)** Top 20 KEGG pathways significantly enriched in the MEbrown module, as identified by WGCNA. The vertical axis represents KEGG pathways and the horizontal axis indicates the significance level of enrichment. The size of dots represents the number of genes and the color of dots represents the *P*-adjust.

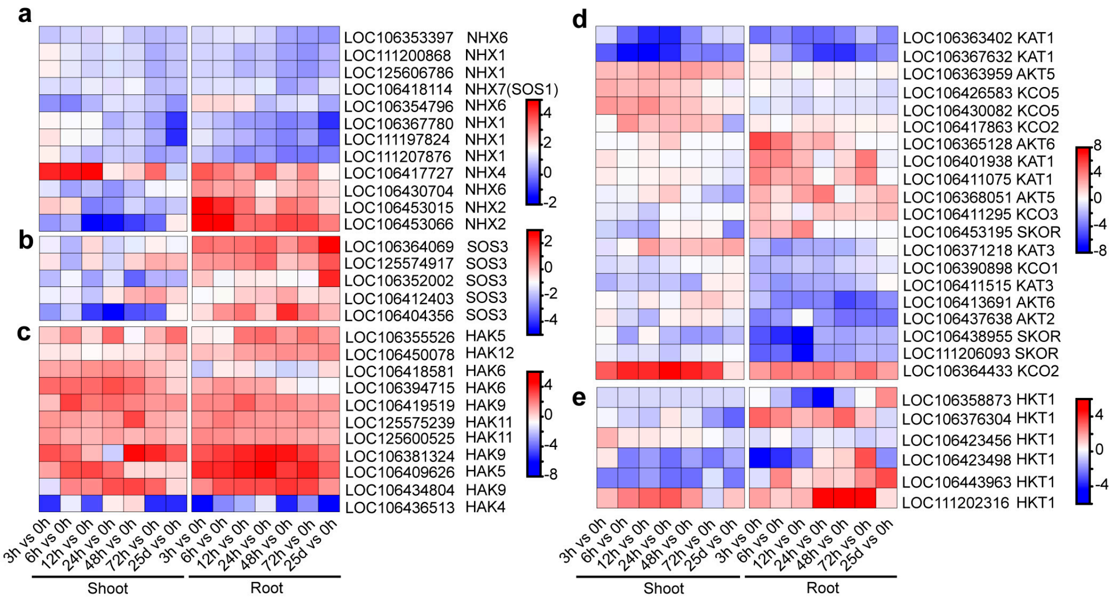

Figure S7. Expression profiles of ion transporter genes. (a-e) show genes of *NHX* family **(a)**, *SOS3* **(b)**, and potassium transport related members *HAK*s **(c)**, *ATK*s, *SKOR*s and *KCO*s **(d)**and *HKT1* **(e)**.

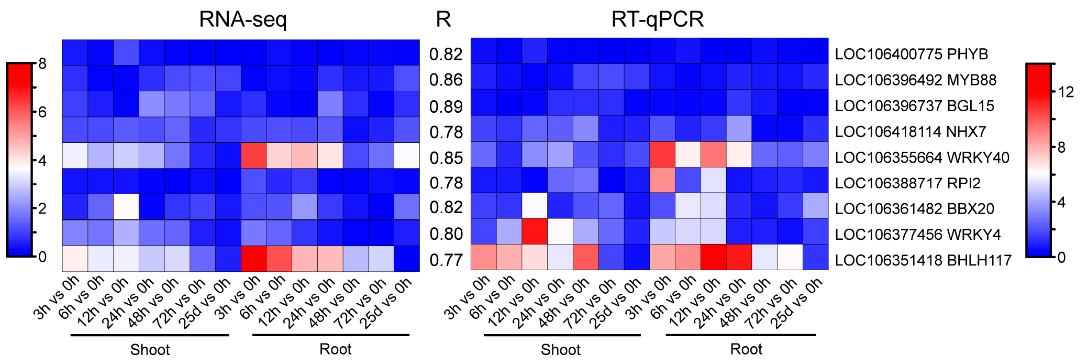

Figure S8. Correlation between expression profiles of selected genes obtained from RNA-seq and RT-qPCR analysis. Heatmaps represent expression profiles of selected genes (labelled on right side) obtained from RNA-seq (left) and RT-qPCR (right) analysis. The color scale at the bottom represents the value of log2|FC|. The values between the two heatmaps represent correlation value between the expression profiles obtained from RNA-seq and RT-qPCR analysis for each gene.
